# Satisfaction With Facial Aesthetic Appearance Following Maxillomandibular Advancement (MMA) for Obstructive Sleep Apnea (OSA): A Meta-Analysis

**DOI:** 10.7759/cureus.35568

**Published:** 2023-02-28

**Authors:** Basem T Jamal, Elaf A Ibrahim

**Affiliations:** 1 Oral and Maxillofacial Surgery, King Abdulaziz University, Jeddah, SAU; 2 Oncology, Newcastle University, Newcastle, GBR

**Keywords:** satisfaction rates, airway obstruction, jaw corrrction surgeries, facial esthethic surgery, obstructive sleep apnea (osa)

## Abstract

Purpose: A large cohort of patients diagnosed with obstructive sleep apnea (OSA) require surgical intervention, sometimes in the form of maxillomandibular advancement (MMA), to correct their functional disturbance. Such a surgical procedure typically results in a slight modification of the patients' facial appearance. The purpose of the current systematic review and meta-analysis was to examine the rate of satisfaction with facial aesthetics post-MMA intervention and to assess its dependability on and relationship with other patient or treatment factors. Based on the literature currently available, and to the best of our knowledge, this is the first paper to draw on the topic analytically.

Methods: A search was conducted on four electronic literature databases (Pubmed, Ovid, Science Direct, and Scholar). Using referred Reporting Items for Systematic Reviews and Meta-Analyses (PRISMA), our inclusion criterion covered any case with adequate reported data pertaining to the research question up to June 2021. Three evaluator groups were utilized. Satisfaction was defined as either an obvious reported increase in fondness for facial appearance or a state of indifference to the cosmetic results of the conducted changes. Dissatisfaction was defined as a clear discontent with the post-operative esthetic results. A multivariate analysis of the data was conducted, and Chi-square tests for independence were used to detect any significant associations. A meta-analysis of proportion was employed to permit for Freeman-Tukey double arcsine transformation and stabilize the variance of each study’s proportion. Cochran’s Q was computed, and the significance level was gauged as a function of P value.

Results: Meta-analyses of proportion conducted for assessment of aesthetic appraisal following surgical MMA for OSA elucidated a significantly higher predilection towards aesthetic satisfaction after surgical MMA for OSA for all evaluator groups in the encompassed studies. 94.2% of patients were satisfied with their facial esthetics postoperatively.

Conclusion: The vast majority of patients that undergo MMA for the correction of OSA report satisfaction with post-surgical facial aesthetics. The subjective assessment of this parameter by physicians and laypeople portrays an equivalently significant skew toward post-surgical appearance improvement. MMA is a generally safe procedure that substantially contributes to enhancement of both overall quality of life and perceived aesthetic appeal.

## Introduction and background

Introduction

Obstructive sleep apnea (OSA) is a disorder involving episodes of partial (hypopnea) or complete (apnea) reductions in breathing during sleep. The obstruction is typically caused by inordinate, intermittent relaxation of the pharyngeal muscles, thus excessively narrowing or completely blocking the airway [1]. This functional disturbance hampers normal breathing and significantly interrupts healthy sleeping patterns.

The incidence of OSA is 2-4% in quadragenarian males [2], 1-2% in tricenarian females [3], and a collective 5-25% in adults above the age of 18 years [4,5]. OSA in adults is associated with multiple adverse health hazards, including serious cardiovascular, neurological, and endocrine consequences. Long term untreated disease can thus significantly impact overall health and quality of life [6]. 

MMA has been successfully applied to treat patients with OSA who have either not responded to non-invasive therapies or were unable to comply with them. Several investigators have reported success rates ranging from 85% to 95% after MMA [7]. The proposed mechanism of correction involves stretching the oropharyngeal soft tissues, which produces a larger posterior airway space [8]. To achieve this level of success, large surgical movements are required, generally on the order of 10 mm [9].

With such advancements in the facial skeleton, the soft tissues will rationally automatically follow this skeletal advancement, up to 90% in the anteroposterior dimension alone. Depending on a patient’s demographics and background, these changes can be viewed as either advantageous or unfavorable. This dictates the importance of preoperative planning and the necessity for discussing predictions of soft tissue changes with the patients beforehand [10]. Functional outcomes do not correlate with facial esthetics [11,12], and therefore postoperative facial appearance is often a limiting concern for patients whose symptoms would otherwise benefit from MMA. 

The purpose of the current systematic review and meta-analysis was to examine the rate of satisfaction with facial aesthetics post-MMA intervention and to assess its dependability on and relationship with other patient or treatment factors. Based on the literature currently available, and to the best of our knowledge, this is the first paper to draw on the topic analytically.

Materials and methods

In this paper, satisfaction was defined as either an obvious reported increase in fondness for facial appearance or a state of indifference to the cosmetic results of the conducted changes; where present, indifference was also separately identified. Dissatisfaction was defined as a clear discontent with the post-operative esthetic results. Several of the included studies addressed the question of esthetics from multiple angles over a series of patients, but most reported cases with clearly defined satisfaction cut-offs. 

Study Identification And Selection

**Figure 1:**
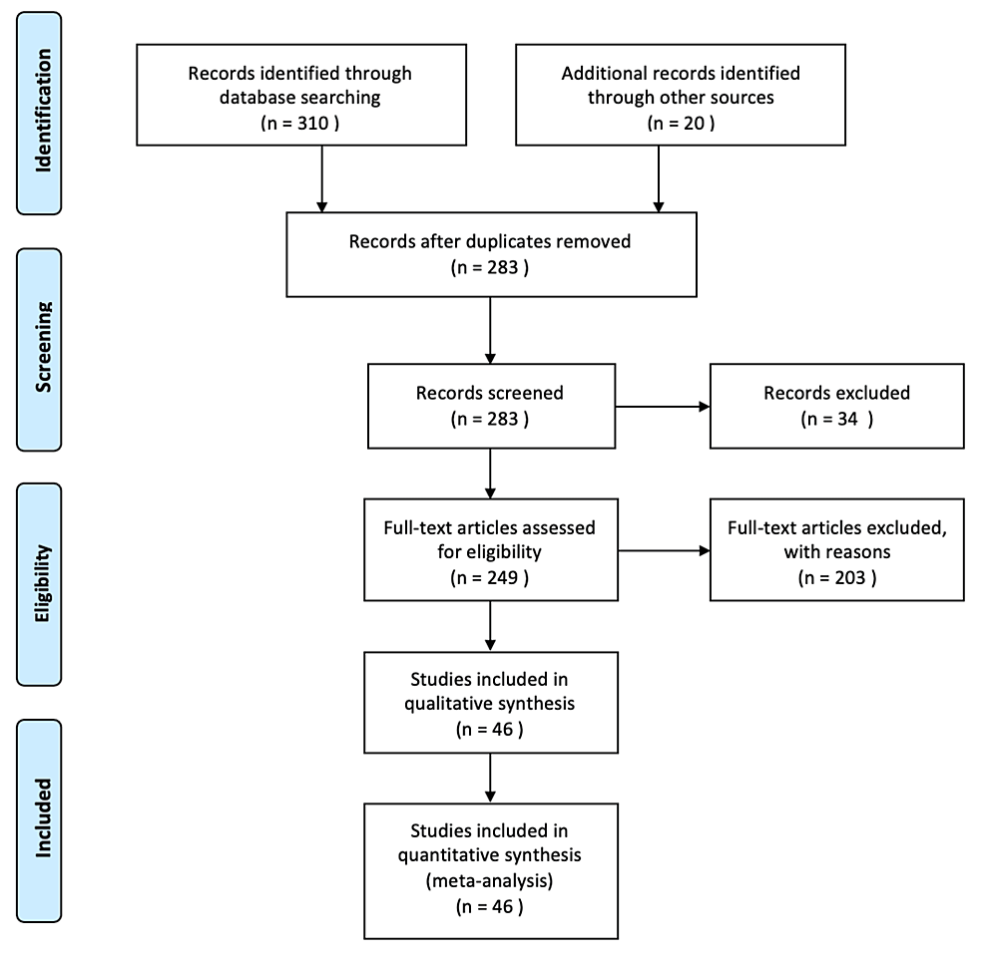
Prisma Flowchart Preferred reporting items for systematic reviews and meta-analysis (PRISMA) flowchart

We conducted a search on four electronic literature databases (Pubmed, Ovid, Science Direct, and Scholar) elected to deliver comprehensive coverage of the available literature. The search was neither bound by date nor language restrictions and incorporated any paper with the keywords of “Maxillo-mandibular advancement/MMA”, “Obstructive sleep apnea/OSA”, and “Esthetic/Cosmetic”. Our inclusion criterion was any case with adequate reported data pertaining to the research question up to June 2021. Exclusion was performed on a case by case basis for inadequately reported information, duplications, and irrelevant records. We have identified no systematic reviews or meta-analyses addressing the satisfaction of adult OSA patients with post therapeutic MMA facial appearance. A Preferred Reporting Items for Systematic Reviews and Meta-Analysis (PRISMA) flowchart [13] is displayed as a summary of our search and review process (Figure 1).

Data Extraction 

From each study, the following data was extracted and tabulated per individual patient case: (a) age, (b) gender, (c) ethnicity, (d) BMI, (e) AHI pre-OP, (f) AHI post-OP, (g) pre-OP orthodontic treatment status, (h) Skeletal class, (i) Maxillary advancement, (j) Mandibular advancement, (k) additional surgical procedures undergone and (l) Esthetic evaluation. The latter was subdivided into patient, physician, and layperson responses. As reporting varied, each study was carefully inspected for these details, and numerical figures were calculated separately, as available. A uniform extraction rubric and success definition was applied to all studies for the purpose of this review. 

Statistical Analysis 

Following data extraction, a randomized sampling technique of 25% of the studies was checked by both authors for accuracy. A multivariate analysis of the data was conducted, and Chi-square tests for independence were used to detect any significant associations. Both random-effect and fixed-effect models were utilized to estimate the pooled satisfaction proportion, with a 95% confidence interval. A meta-analysis of proportion was employed to permit for Freeman-Tukey double arcsine transformation and stabilize the variance of each study’s proportion. This also served to apply continuity correction to evade exclusion of studies with 100% or 0% satisfaction. Each category of evaluators was analysed separately to avoid assessment bias.

Heterogeneity between the included studies was assessed and measured. Cochran’s Q was computed, and the significance level was gauged as a function of P value. The I2 statistic, describing variation percentage across studies due to heterogeneity rather than chance, was also calculated. Statistical analysis was conducted using SPSS version 27.0.1.0 (IBM Corp., Armonk, NY) and MedCalc version 20.115 (MedCalc Software bv, Ostend, Belgium). 

## Review

Results

*Demographics* 

**Figure 2:**
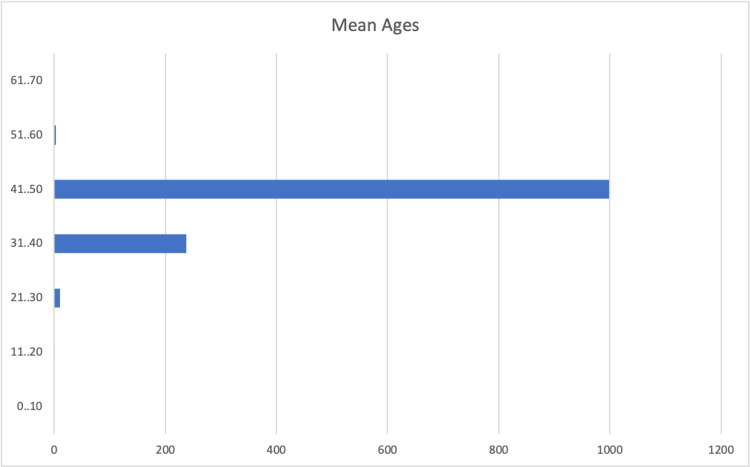
Mean ages Graphical representation of the patients’ mean ages across the included studies

From the included 46 papers [10-12,14-57], a total number of 1268 eligible patient records were obtained. 80.9% (n= 1026) of the patients were males, 13.8 % (n= 176) were females, and 5.3% (n=62) were of an unspecified gender. The ages, calculated for 1215 patients, ranged from 21-71 with a mean of 42.7 years (Figure 2). The largest proportion of patients belonged to the 41-50 years age group. The average preoperative BMI, calculated for 867 patients, was 27.9 kg/m^2^ with a range of 17.3-48.8 kg/m^2^. The Apnea-Hypopnea Index (AHI) preoperatively ranged from 8.2 to 121 and averaged 46.8 events per hour, calculated for 614 patients. The vast majority of patient reports, 90.2% (n=1144) had no record of their ethnicity described, 7.49% (n=95) of the patients were Chinese, 1.57% (n=20) were Taiwanese, 0.86% (n=11) were White, 0.23% (n=3) were Malay, 0.07% (n=1) was Eurasian, and 0.07% (n=1) was Indian.

*Surgical Data* 

The mean maxillary advancement performed, calculated for 498 patients, was 5.8 mm, and the mean mandibular advancement, calculated for 520 patients, was 8.1 mm. When present, rotation was mostly in the counterclockwise direction; only in two instances have authors reported clockwise rotation. Skeletal classification of patients preoperatively was not reported for 69.4% (n=881); 7.2% (n=92) of patients were class I, 19.3% (n=245) were class II and 4.5% (n=57) were class III. Postoperatively, the AHI averaged 9.62 and ranged from 0-86 events per hour, calculated for 579 patients. Of the total, only 82 patients were reported to have undergone pre-operative orthodontic treatment.

**Figure 3:**
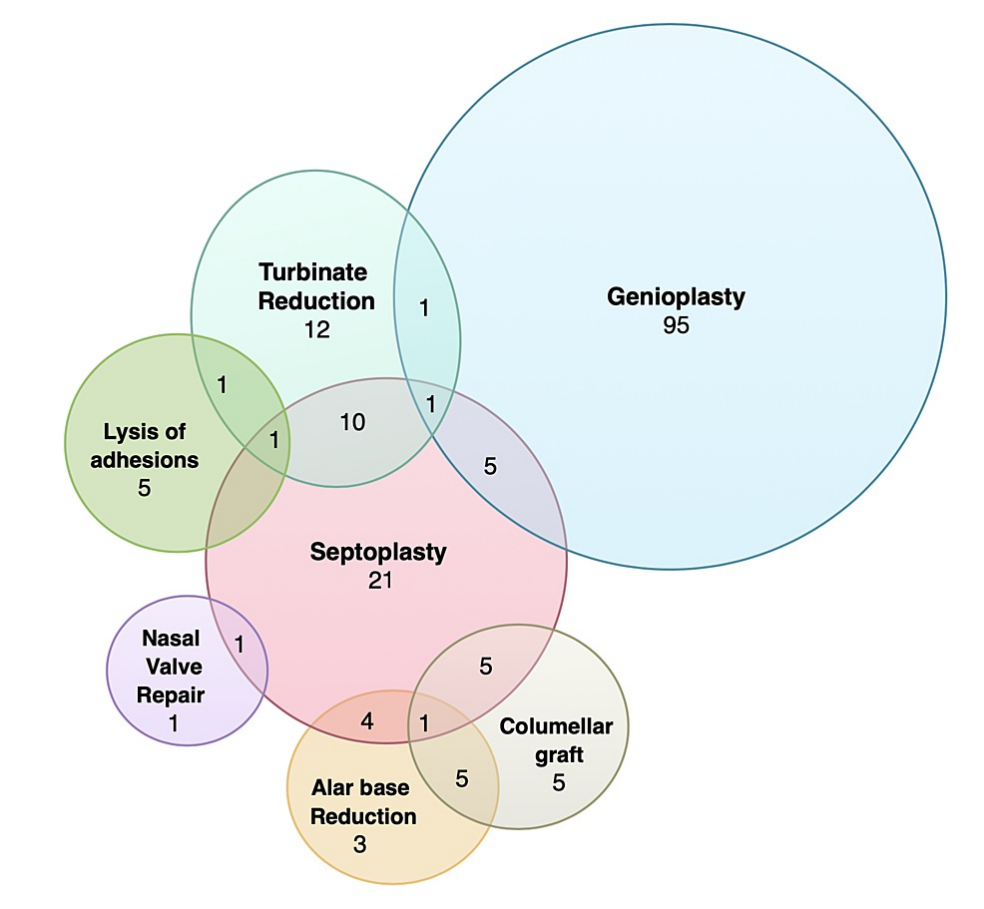
Additional surgical procedures Venn diagram illustrating the additional surgical procedures undergone by patients either prior to or concomitantly with MMA. *Data for patients with reported esthetic satisfaction only* MMA: Maxillomandibular Advancement

Collectively, 32.2% (n=408) patients have undergone additional surgical procedures, 512 procedures in total, either prior to or concomitantly with their MMA. Of those 408 patients, 27.7% (n=142) had a genioplasty, 13.7% (n=70) had a uvulopalatopharyngoplasty (UPPP), 21.5% (n=110) had a septoplasty, 16.4% (n=84) had a turbinate reduction, 1.4% (n=7) had lysis of nasal adhesions, 0.4% (n=2) had nasal valve repair, 3.2% (n=16) had a columellar graft, 2.5% (n=13) had an alar base reduction, 5.3% (n=27) had a cervicofacial lipectomy, 2.5% (n=13) had maxillary sinus surgery, 0.4% (n=2) had a tracheostomy, 3.9% (n=20) had an anterior segmental osteotomy, 0.4% (n=2) had a hyoid suspension and 0.7% (n=4) had a sliding chin osteotomy. One hundred and seventy-seven of the 408 patients had their satisfaction with the facial appearance recorded. Figure 3 depicts those for which satisfaction data was available. 

Satisfaction With Facial Appearance

Satisfaction with the esthetic results of surgery was divided into three categories: (a) Satisfied, (b) Not Satisfied, and (c) Indifferent. It was also assessed from the different angles of three evaluator groups: (i) Patient responses, (ii) Physician responses, and (iii) Laypeople responses. Authors of the original data collected this information through patient questionnaires and surveys of subjective assessment. Physician and Laypeople responses were collected either from direct patient observation and subjective assessment or from evaluation of patient photographs or silhouettes. 

**Figure 4:**
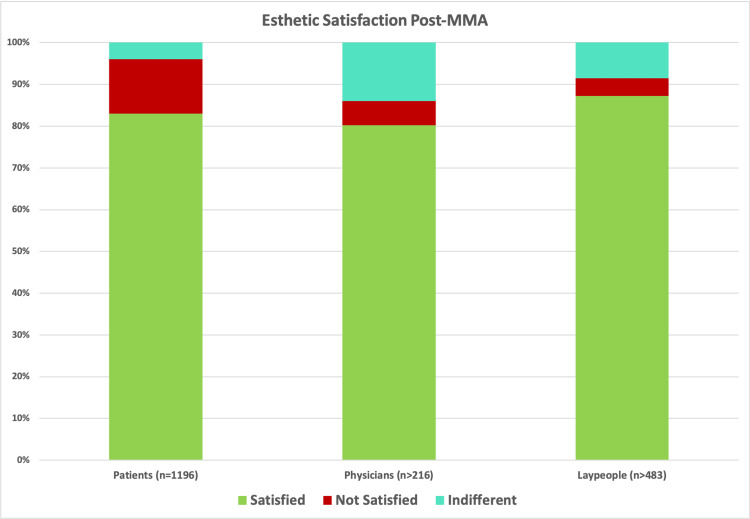
Aesthetic satisfaction scores Stacked bar chart illustrating satisfaction of all three evaluator groups with post-MMA facial esthetic results

In total, 1196 patient responses, physician evaluations of 171 patients, and layperson evaluations of 82 patients were collected. 85.6 % (n=1024) of patients were satisfied with their facial esthetic results postoperatively, 8.6% (n=103) were indifferent, and 5.8% (n=69) were not satisfied. Physicians evaluated 94.2% (n=161) of patients to have satisfying cosmetic appearances after surgery, observed no difference in appearance in 2.9% (n=5), and described a decline in attractiveness in 2.9% (n=5). Laypeople appraised 73.2% (n=60) of patients to have satisfying esthetic changes after surgical intervention, detected no difference in appearance in 12.2% (n=10), and noted less satisfying postoperative looks in 14.6% (n=12). These results are summarized in Figure 4.

Comparative Statistical Analysis 

Contingency tables showed no significant correlation between the factors of age and evaluator responses. This was also noted for correlation of the latter with gender. Understandably, patients with a preoperative skeletal class II and III reported more marked esthetic satisfaction post-MMA than patients with preoperative skeletal class I. Patients who have undergone preoperative orthodontic treatment showed similar satisfaction report patterns as those who have undertaken only surgical interventions. 

Contrary to primary speculation, no significant associations were observed between satisfaction assessments and racial background either. This was true even for the included Chinese patients. Chinese people recognizably have more convex facial profiles and protrusive lips compared to Africans and Caucasians. Consequently, the likelihood of acquiring less acceptable esthetic alterations following displacement of the maxillo-mandibular complex is generally higher [14]. This risk is pronounced in patients with thin facial soft tissues which fail to mask large maxillary advancements [15]. However, statistical analysis has shown that of the indicated 95 evaluations of Chinese patients, only 7% reported dissatisfaction with esthetic surgical results.

Esthetic satisfaction was also higher in patients with more complex preoperative functional disturbances. Individuals with AHIs of 20 or higher were more satisfied with surgical outcome than those with AHIs of less than 20. This trend was consistent across the evaluations of physician and laypeople groups as well. Mandibular advancements of less than 5 mm rendered higher esthetic satisfaction among the patient evaluators. Maxillary advancements showed an analogous pattern, with movements of less than 5 mm resulting in higher subjective patient gratification. However, no difference in perceived esthetic acceptance was noted between results of less than or more than 5 mm for cases assessed by physicians and laypeople. 

Satisfaction with facial appearance was also appraised in light of surgical outcome. Surgical treatment success was defined relative to Sher’s success criteria, outlined as a minimum of 50% reduction in the apnoea-hypopnea index and a concomitant translation into less than 20 respiratory obstructive events per hour [16]. Significantly, aesthetic satisfaction reported by patients and physicians was satisfactory following MMA regardless of functional surgical outcome in the management of OSA.

**Figure 5:**
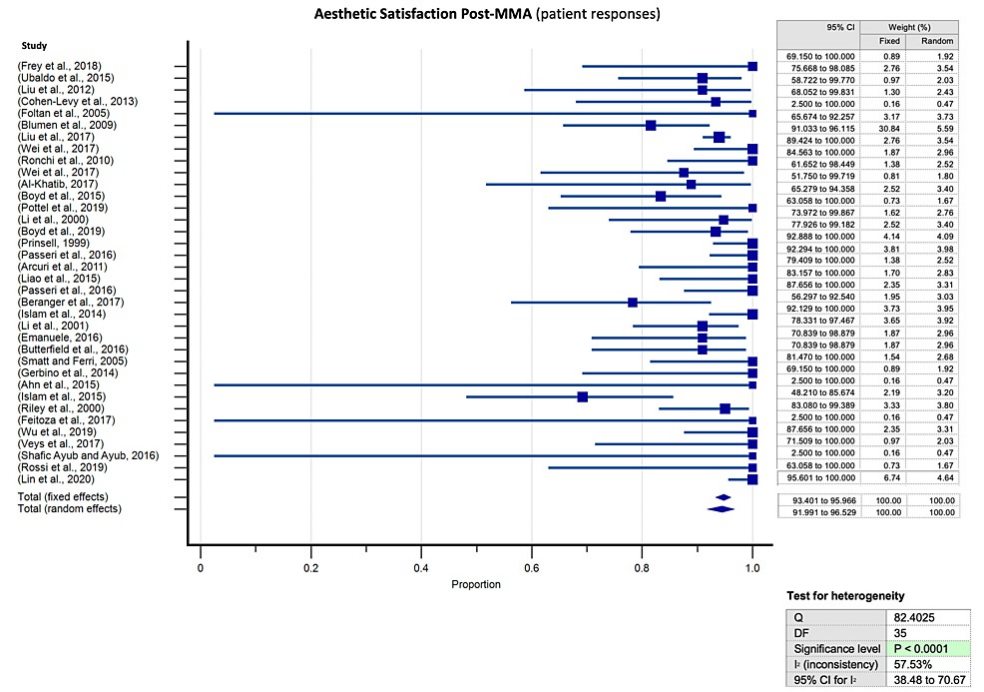
Meta-analysis of proportion for satisfaction (patient satisfaction) Meta-analysis of proportion for satisfaction and 95% confidence interval (CI) by surgical outcomes as evaluated by subjective patient assessments, with relevant heterogeneity testing values.

**Figure 6:**
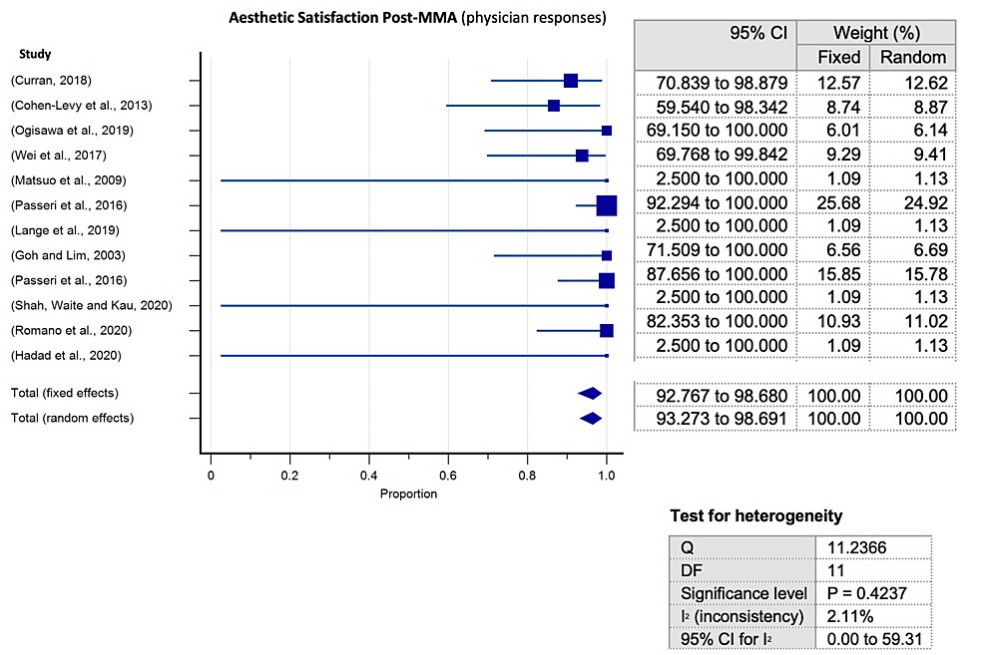
Meta-analysis of proportion for satisfaction (physician satisfaction) Meta-analysis of proportion for satisfaction and 95% confidence interval (CI) by surgical outcomes as evaluated by physician assessments, with relevant heterogeneity testing values

**Figure 7:**
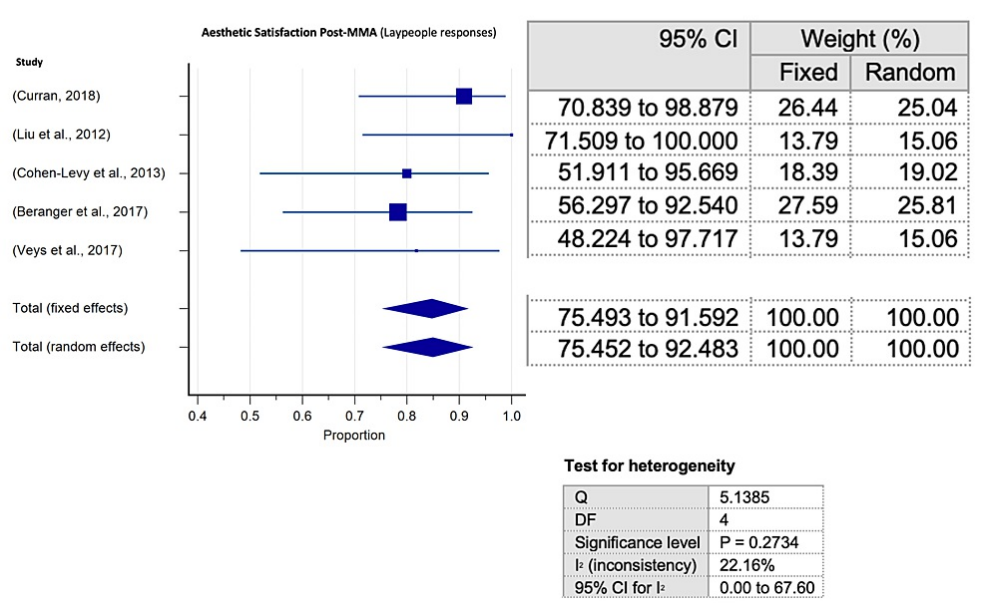
Meta-analysis of proportion for satisfaction (laypeople satisfaction) Meta-analysis of proportion for satisfaction and 95% confidence interval (CI) by surgical outcomes as evaluated by laypeople assessments, with relevant heterogeneity testing values.

Meta-analyses of proportion conducted for assessment of aesthetic appraisal following surgical MMA for OSA elucidated a significantly higher predilection towards aesthetic satisfaction after surgical MMA for OSA for all evaluator groups in the encompassed studies. Articles tackling subjective patient evaluations of facial appearance were significant for substantial heterogeneity (p< 0.0001) (Figure 5). Heterogeneity was not significant neither for articles assessing physician evaluations (Figure 6) nor for those evaluating laypeople judgements (Figure 7).

Discussion

As important as its physiological function is, the face is primarily considered an organ of identity. The relationship between appearance and self-image is well documented in the literature. Given so, one’s self-worth is closely intertwined with how he is perceived by himself and others [56]. Understandably, any alteration to what is observed as normal or pleasing can have profound psychosocial implications. It is thus that cosmetic factors of the maxillofacial region are the most complex to handle.

MMA for OSA is intended to increase the pharyngeal volume and the airway dimension; however, the facial soft tissue and hence the appearance will also respond to the skeletal movements of the jaws. Numerous reports studied soft tissue to hard tissue ratios in patients undergoing skeletal jaw advancements, with most studies demonstrating ratios of nearly 1:1 [58,59]. With large maxillary advancements, such as in MMA for OSA, unesthetic changes can be anticipated including significant upturning of the nasal complex and considerable fullness of the nasolabial region [60]. While patients seeking treatment for OSA do not desire esthetic facial improvement, they do ask about the potential change in appearance and whether it will lead to deformity, which is an important consideration in their decision to agree to this treatment modality.

While favorable and more attractive ratings have been reported with MMA, even when no underlying dentofacial deficiencies were present [28], a proportion of dissatisfied patients has been reported in the literature ranging from 9 to 31%, who were unhappy with their postoperative appearance [34]. Beauty standards vary across cultures, however, a pronounced jawline and prognathic mandible is a beauty trend now and considered an attractive feature for both males and females. While MMA accomplishes that, it’s also considered the most effective procedure to achieve what is referred to as “reverse facelift” through the skeletal advancement, which will also stretch the muscles, tissues, and tendons if a forward direction enhancing the facial appearance [48].

Our study showed that the vast majority of patients showed improvement in facial appearance following the MMA, whether judged by the patients, physicians or lay people. Patients with preoperative craniofacial skeletal deformities were more pleased with the surgical aesthetic results [30]. The majority of data collected was based on patient responses to their evaluation of the facial appearance, and only 5.8% were not satisfied with the remaining 94.2% either satisfied or indifferent and so the facial appearance was not a factor of significance for them. Even among patients with reported dissatisfaction with their facial appearance, the percentage of individuals who disclosed that they would recommend MMA to other patients was significant. This illustrates that, despite the unpleasant change in appearance, the functional and health related benefit from the surgery outweighed any perceived negative change in facial esthetics [25].

Furthermore, even among surgeries that did not meet the success criteria for the management of OSA, all patients reported indicated satisfaction with their facial appearance despite no improvement in their OSA and this was significant for both patient responses and physician evaluations. In conclusion, MMA for OSA rarely affects the patients perceived facial appearance in a negative way. On the contrary it leads to improved facial appearance for the majority of patients.

## Conclusions

The vast majority of patients undergoing maxillo-mandibular advancement for the correction of obstructive sleep apnea report satisfaction with post-surgical facial aesthetics. The subjective assessment of this parameter by physicians and laypeople portrays an equivalently significant skew toward post-surgical appearance improvement. MMA is a generally safe procedure that substantially contributes to enhancement of both overall quality of life and perceived facial aesthetic appeal.
